# Dietary diversity practice and associated factors among adolescents in Ethiopia, systematic review and meta-analysis

**DOI:** 10.1371/journal.pone.0284573

**Published:** 2023-07-28

**Authors:** Fanos Yeshanew Ayele, Aregash Abebayehu Zerga, Fikre Bayu Gebeyehu, Sisay Eshete Tadesse, Segenet Zewude, Yeshi Habteslasie, Zena Befekadu

**Affiliations:** 1 School of Public Health, College of Medicine and Health Sciences, Wollo University, Dessie, Ethiopia; 2 Department of Anatomy, School of Medicine, College of Medicine and Health Science, Addis Ababa University, Addis Ababa, Ethiopia; 3 School of Pharmacy, College of Medicine and Health Sciences, Wollo University, Dessie, Ethiopia; 4 Department of Agro Economics, College of Agriculture, Wollo University, Dessie, Ethiopia; 5 Department of Rural Development and Agricultural Extension, College of Agriculture and Natural Resource, Mekdela Amba University, Tuluawlya, Ethiopia; Universidade de Sorocaba, BRAZIL

## Abstract

**Background:**

Adolescence (10–19 years) is a critical life period for growth and health. An increase in dietary diversity in the food is related to improved nutrient adequacy of the diet. However, studies conducted on dietary diversity practice among adolescents showed non-conclusive and inconsistent findings on the magnitude of the problem. Likewise, there was no meta-analysis conducted in the study area. Hence, this systematic review and meta-analysis were conducted to estimate the pooled prevalence of good dietary diversity practice and its associated factors among adolescents in Ethiopia.

**Methods:**

The Preferred Reporting Items for Systematic Reviews and Meta-Analyses guideline was followed. All articles were systematically searched by Google Scholar, PubMed, Hinari, Cochrane Library, Global Health and CINAHL. Meta-analysis was conducted by using STATA 14 software. Forest plots were used to present the pooled prevalence of good dietary diversity practices. A random effect model was used to compute the pooled prevalence; while subgroup analysis was performed to identify the possible source of heterogeneity. Publication bias was assessed by the begg’s and egger’s test. Moreover, the associated factor of dietary diversity practices was observed.

**Results:**

This review involved 7 studies, and 3,950 participants. The pooled prevalence of good dietary diversity practice among adolescents in Ethiopia was 39.24% (95% CI: 30.82, 47.66). Mothers with formal education [AOR = 1.98, (95% CI: 1.65, 2.36)], fathers with formal education [AOR = 2.30, (95% CI: 1.81, 2.93)], Medium wealth index [AOR = 2.75, (95% CI: 1.96, 3.86)] and urban residence [AOR = 2.88, (95% CI: 1.59, 5.22)] were positively associated with good dietary diversity practice.

**Conclusions:**

The pooled prevalence of good dietary diversity practices among adolescents is low. Being urban residents, the medium wealth quintile, mothers’ educational status and fathers’ educational status were independent factors of good dietary diversity practice among adolescents. Therefore, focused nutritional interventions should be given to rural residents and adolescents from low economic status.

## Background

Dietary diversity (DD) is defined as the number of food items or food groups consumed in a given period by an individual to insure diet variety, diet quality, and nutrient adequacy. Dietary diversity scores (DDS) are calculated by summing either the number of foods or food groups eaten over a location period An increase in DDS is related to increased nutrient adequacy of the diet [1].

Adolescence (10–19 years) is a crucial life period for growth and health. This age group could be the last opportunity to prevent stunting, the parents of the future; their nutritional status shapes the health of the next generation. Globally, over 1.2 million peoples were in the age group of adolescents, and around 26, 475(24%) of the Ethiopian population were adolescents [2].

In Low and Middle Income Countries, only 17% of adolescents consumed adequately diversified diet [3]. The magnitude of adequate diversified diet feeding practice among adolescent was 23.5–61.4% in Iranian [4, 5], 53% in Nigeria [19] and 17.6–56.7% in Ethiopian [6, 7].

Globally, in a resource-poor setting, inadequately diversified food or low-quality repetitive diets are the norms. Mostly grain- or tuber-based staple foods groups dominate other food groups (vegetables, fruits, and animal-source foods). This type of feeding is exposed to a variety of micronutrient deficiencies [8]. Malnutrition during early adolescent age has long-lasting consequences which affect negatively the overall growth, cognitive development, morbidity, educational performance and productivity [9].

However, adolescents are very susceptible to major economic and social changes, with causing behaviours that threaten health and change their dietary habit. Adolescents are usually open to innovative ideas; they show concentration and interest. Changes in lifestyle, including food habits, are often more apparent among urban adolescents. Therefore, they are typically the ‘early adopters’ owing, among other things to their attraction for innovation and high exposure to commercial marketing [10]. Under nutrition among adolescents has a strong linkage with poor household socioeconomic condition, burden of disease and unequal intra-familial distribution of food, cultural beliefs, mass media, and body image perception [11].

A different study revealed that higher family education, annual income [12–14], food insecure households, adolescents’ educational status [14, 15], female-headed households, and poor household wealth [14] were the main contributing factors for the dietary diversity practice of adolescents.

Usually in developing countries nutrition interventions have been the focus on children and women. Addressing the nutrition requirements of adolescents could be a significant step to break the vicious cycle of chronic diseases, poverty, and intergenerational malnutrition [16].

Determining the magnitude of dietary diversity practice among adolescents is vital to designing effective interventions to reduce inadequate dietary diversity practice. There is no nationally representative data that provides an estimate of dietary diversity practice in Ethiopia. Even though dietary diversity practice was previously studied in Ethiopia, the findings of the studies were varying and there was no single conclusive finding regarding the prevalence and factors affecting dietary diversity practice in Ethiopia. In addition, prior systematic reviews and meta-analyses have not been conducted and public health experts and policymakers who are working with adolescents need updated evidence regarding dietary diversity practice. Therefore, this review aimed to estimate the pooled prevalence of good dietary diversity practices among adolescents in Ethiopia.

## Materials and methods

### Registration

The preferred reporting items for systematic review and meta-analysis (PRISMA) guidelines were strictly used to perform this systematic review and meta-analysis [17]. It has been registered in the International Prospective Registry of Systematic Review (PROSPERO) with a specific registration number of CRD42021248524.

### Search strategy and studies identification

The studies were retrieved compressively from both published and unpublished articles. The published articles were searched by using different databases such as PubMed, Cochrane Library, CINAHL, HINARI library, Web of Science, Scopus, Science Direct Food Science and Global Health and unpublished literature sources like Google Scholar, and Google was explored. searched by All relevant articles were retrieved by using a combination of search terms/ keywords like; “dietary diversity practice”, “inadequate dietary diversity”, “low dietary diversity”, “determinant”, “Predictors”, “factors”, “associated factors”, “adolescent”, “female adolescent”, “Ethiopia’‘ using Boolean operators "OR" or "AND" as appropriate and the search was done by two authors independently FY, AA, SE, SZ, ZB, YH and FB).

### Inclusion and exclusion criteria

All studies irrespective of data collection and publication year until the end of march 1, 2022 were included in this review. This review included published and unpublished studies that were conducted on the dietary diversity practice among adolescents in Ethiopia. All observational studies with English language publications which measured the dietary diversity practice; inadequate dietary diversity score, low dietary diversity score, and adequate dietary diversity practice among adolescents were included in this review. However, pure qualitative studies, studies with poor methodological quality, and studies in which the outcome was not reported were excluded from the review.

### Outcome variable

Dietary diversity practice is the variety of foods consumed by adolescents out of ten food groups. These food groups include starch staples, vitamin A-rich vegetables and fruits; dark green leafy vegetables; other vegetables; other fruits; meat, poultry, and fish; eggs and pulses/legumes; nuts and seeds; and dairy products [18]. Good dietary diversity is considered adolescents consume five or more varieties of food groups used in this study. The primary outcome of this review was the prevalence of good dietary diversity practices among adolescents. The second objective was the associated factors of good dietary diversity practices conducted in Ethiopia. The odds ratio/ 2x2 contingency was extracted for associated factors of good dietary diversity practice.

### Study selection, risk of bias assessment, and data extraction

Those studies searched from selected databases were transferred to End note, and duplicate files were excluded. The rest articles and abstracts were separately screened by two groups (FY, FB, SZ, YH and AA) for inclusion in the full-text appraisal. The differences between reviewers were managed by discussion and disagreement was handled by the third party (SE and ZB). The quality of articles was evaluated using the Joanna Briggs Institute (JBI) critical appraisal checklist [19]. Two reviewers separately assessed articles before inclusion for review.

Seven authors (FY, AA, FB, YH, SZ, SE and ZB,) independently retrieved all the necessary data using Microsoft Excel 2010 sheet. The data extraction tool contains information on the author’s name, year of publication, study area, response rate, sample size, study quality score, and prevalence.

### Statistical methods and analysis

The statistical analysis was conducted using STATA 14 software. A Forest plot was used to display the magnitude of dietary diversity practices among adolescents in Ethiopia. Due to the significant presence of heterogeneity among studies, the random effect model of analysis was applied. The pooled prevalence of diversity practice among adolescents was presented with a 95% CI. The heterogeneity test of involved studies was measured by using the inverse variance (I^2^) statistics. The value of I ^2^ was interpreted as 25% as low, 50% as a medium, and 75% as high heterogeneity. For this review, we used p -value less than 0.05 to declared the presentence of heterogeneity [20].

Subgroup and sensitivity analysis was also conducted by different study characteristics such as sub-region of Ethiopia (Amara or other), sample size (small or large), and residence (urban or rural). The presence of publication bias was assessed using Begg’s test and the Egger regression asymmetry test [21]. When the p-value was less than 0.05 declared the presence of a publication bias.

## Results

### Study selection

**Fig 1:**
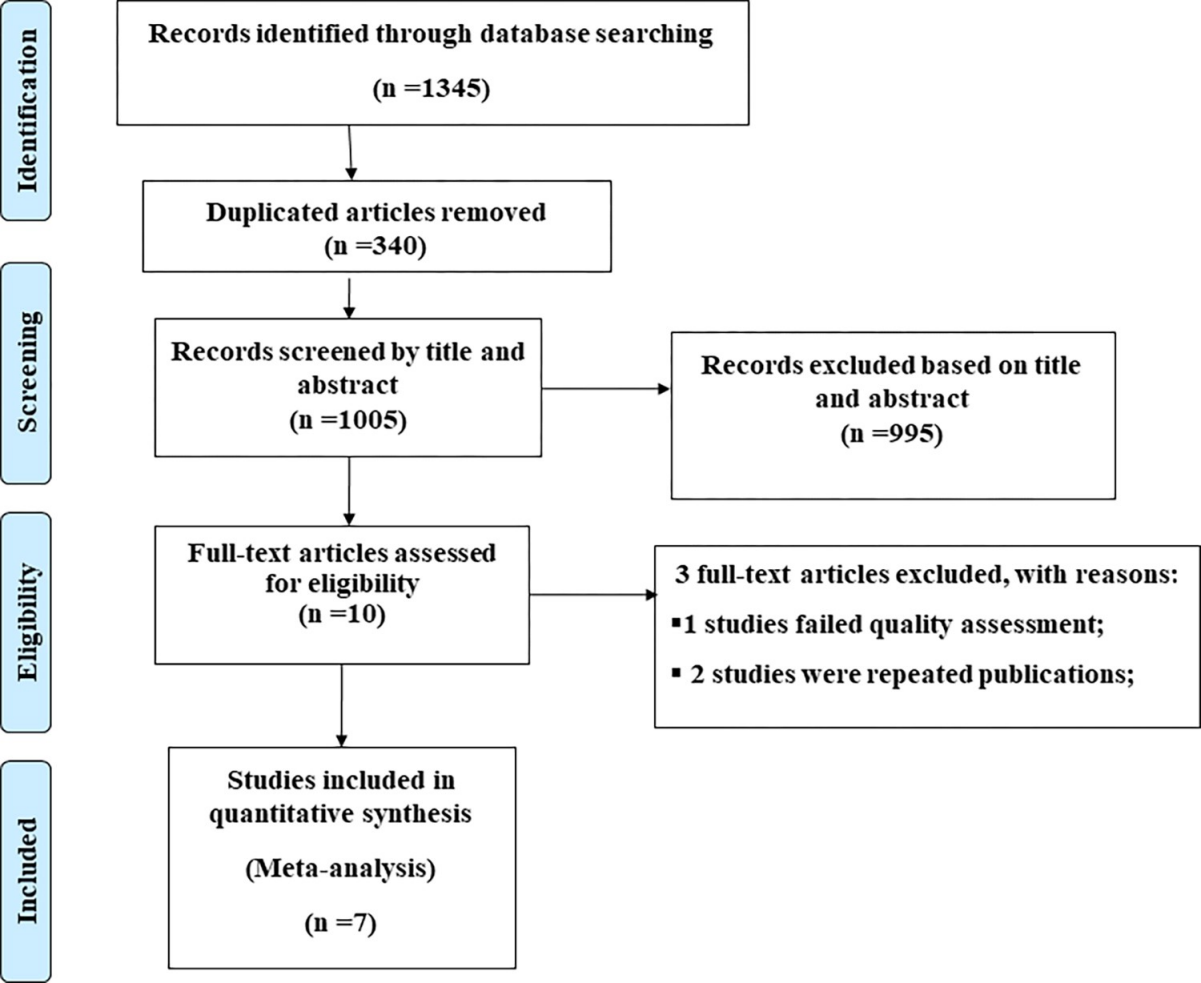
PRISMA Flow diagram of the included studies for meta-analysis of dietary diversity practice and associated factors among adolescents in Ethiopia.

This systematic review and meta-analysis, identify1345 published, and unpublished studies conducted on dietary diversity practice and associated factors among adolescents in Ethiopia. Then 340 were excluded due to duplication and the remaining 995 studies were excluded due to their titles and abstracts. Ten full-text articles were assessed for eligibility. From these, 3 full-text articles were removed for prior criteria, and a total of 7 studies were included in the review (Fig 1).

### Characteristics of included studies

A total of 7 studies [6, 22–27] with 3,950 study participants were included to estimate the pooled prevalence of dietary diversity practice and associated factors among adolescents in Ethiopia. The sample size of the studies ranged from 400 study participants in a study conducted in the Tegede district [22], to 820 participants in a study conducted in Wolaita Zone [6]. In this meta-analysis, three regions from nine regions of the country were represented; four studies from Amhara [22–25], two from SNNP [6, 26] one from Oromia [27]. The studies were conducted from 2015 to 2021 in different regions of the country (Table 1).

**Table 1:** Summary characteristics of studies included in the meta-analysis of the prevalence of dietary diversity practice among adolescents in Ethiopia.

Authors and Publication year	Study year	Study area	Study design	Sample size	Response rate	Prevalence (%)		Quality score
						Adequate dietary diversity practice	Inadequate dietary diversity	
Berhanu et al, 2019	2018	Tegede district	School-based cross-sectional	400	100	47	53	80%
Birru et al, 2018	2017	Gondar City	School-based cross-sectional	768	98.7	52	48	85%
Worku et al, 2017	2016	Gurage	School-based cross-sectional	634	100	26.8	73.2	78%
Halala et at., 2020	2019	Wolaita Zone	Community Based cross sectional	820	97.3	27.6	72.4	68%
Melaku et al., 2017	2015	Jimma town	School based cross sectional	455	100	38.7	61.3	71%
Gonete et al, 2020	2017	Dembia district	School-based cross se 9ik,m ctional	462	97.5	32.3	67.7	82%
Endalifer et al, 2021	2016	Woldia City	School-based cross-sectional	411	97.3	50.9	49.1	75%

### Prevalence of good dietary diversity practice

**Fig 2:**
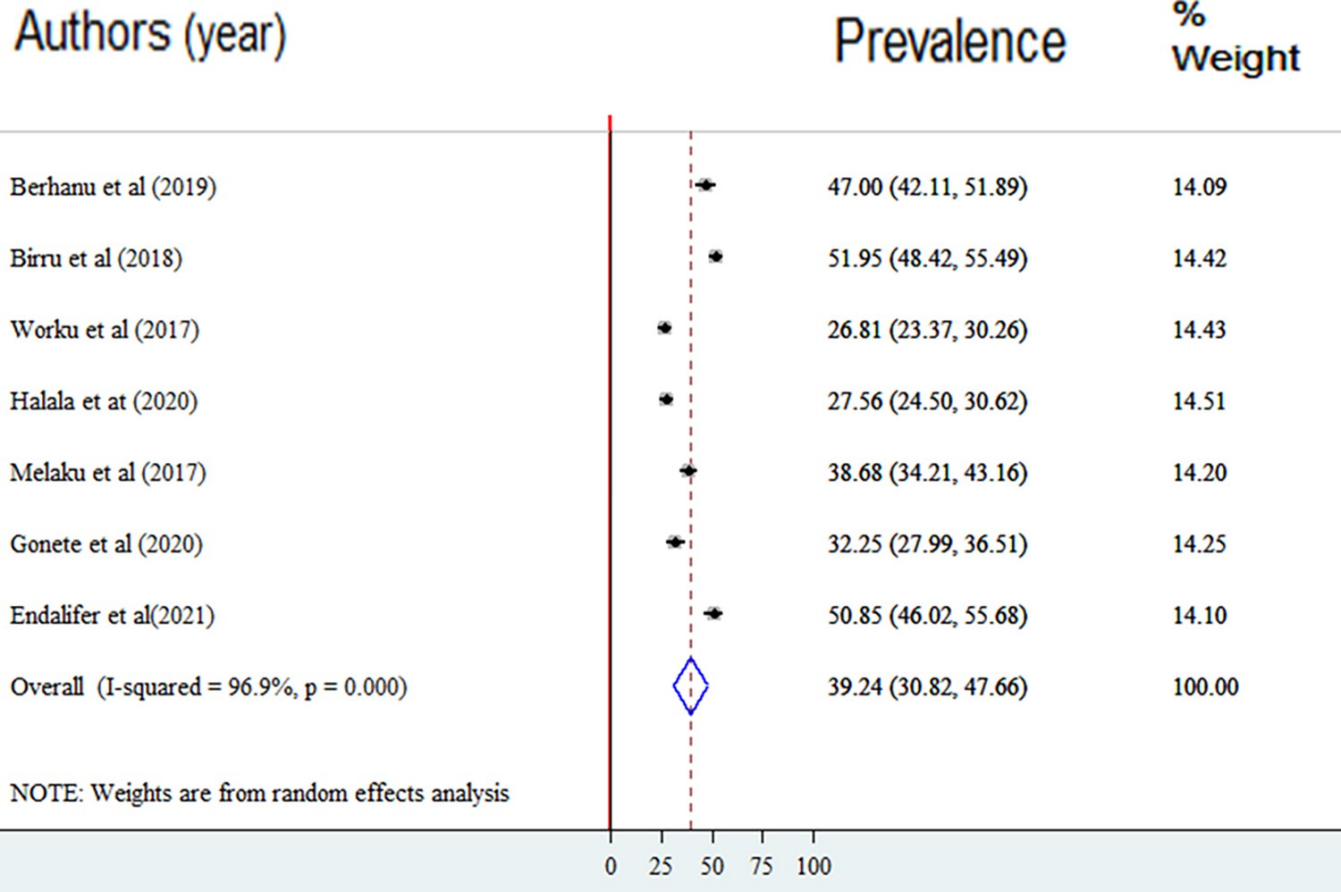
Forest plot of the prevalence of good dietary diversity practice among adolescents within Ethiopia.

**Fig 3:**
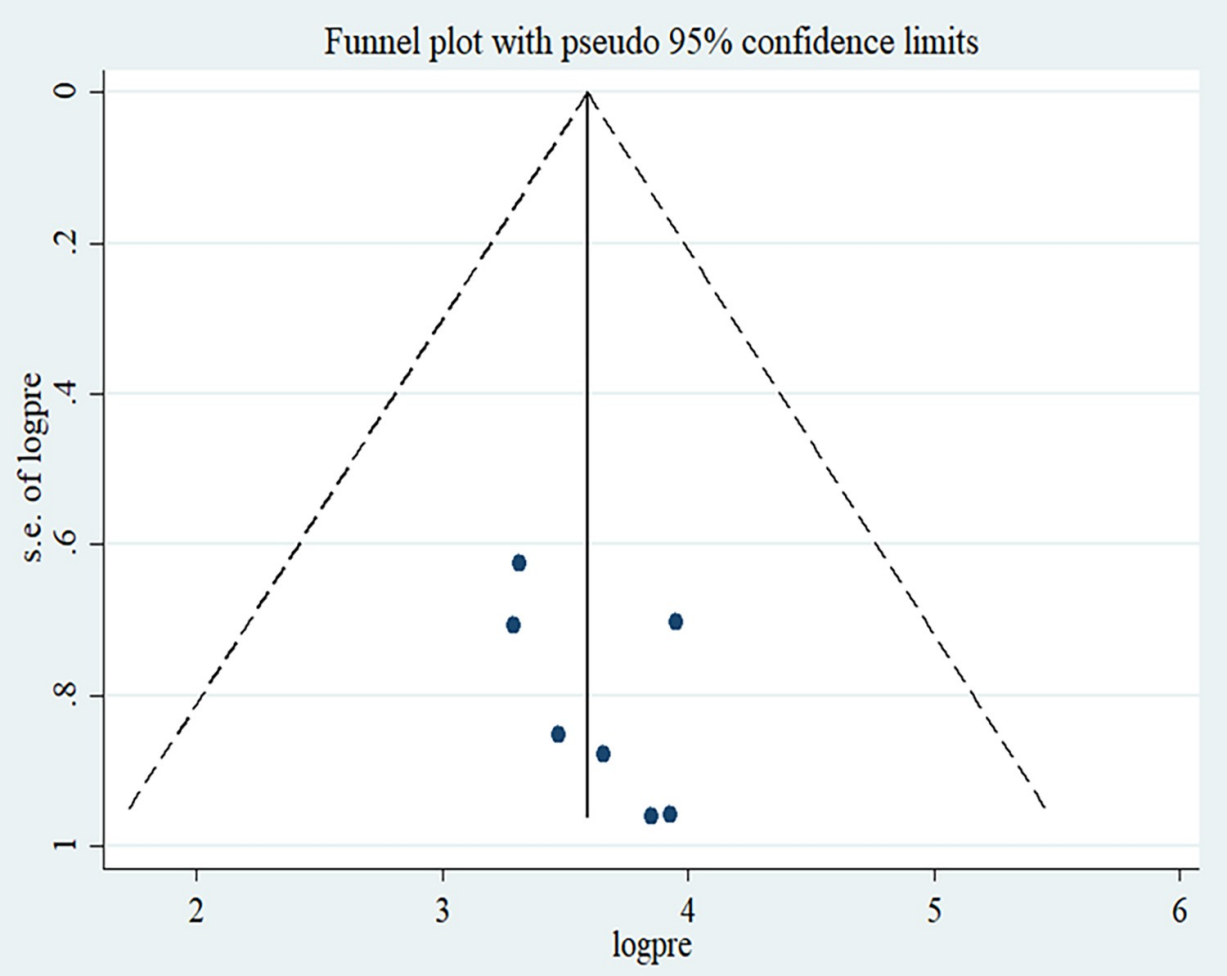
Funnel plot for the prevalence of good dietary diversity practice among adolescents in Ethiopia.

The overall pooled lifetime prevalence of adequate dietary diversity practice among adolescents in Ethiopia was 39.24% (95%CI: 30.82, 47.66). The highest lifetime prevalence of adequate dietary diversity practice was reported in a study done in Gondar city. The study showed that 51.95% of adolescents had experienced adequate dietary diversity practice [25]. The lowest prevalence of adequate dietary diversity practice was 26.81% among adolescents in Guragie Zone [26]. Significant heterogeneity was observed among included studies in the meta-analysis, I 2 = 96.9%, p < 0.001 (Fig 2). The funnel plot showed a symmetrical appearance (Fig 3). The Egger’s regression asymmetry test also showed non-significant publication bias, p-value = 0.21. Sensitivity analysis was conducted to identify the possible source’s source of bias, but no outlier study potentially shifted the primary pooled estimates.

### Subgroup analysis

The subgroup analysis was conducted by region, sample size, residence, and sample size to identify the discrepancy across the individual studies. Even though heterogeneity still existed in the subgroup analysis of all the above-mentioned factors, the prevalence of dietary adequate diversity practice was significantly higher among studies conducted in the Amara region [45.5%, 95% CI: (36.3, 54.8)] as compared to studies conducted in another region. And also, the prevalence of adequate dietary diversity was a significant difference between small sample and large sample sizes [42.1, 95% CI: (33.9, 50.4)] and large sample size [35.4, 95%CI: (19.7, 51.1)] (Table 2).

**Table 2:** Subgroup analysis of the dietary diversity practice among adolescents in Ethiopia.

Sub-groups	Number of studies	Total sample	Prevalence (95% CI)	Heterogeneity	
				I^2^	p-value
By region					
Amara	4	2041	45.52 (36.26, 54.77)	94.5	< 0.001
Other	3	1909	30.84 (24.24, 37.45)	90.0	<0.001
By sample size					
Small	5	1728	42.14(33.85, 50.42)	92.3	< 0.001
Large	2	2222	35.43 (19.72, 51.13)	98.5	< 0.001
By residence					
Both urban & rural	3	2264	39.48 (26.97, 52.00)	97.5	<0.001
Urban	4	1686	38.93 (25.37, 52.50)	97.0	< 0.001
Total	7	3950	39.24 (30.82, 47.66)	96.9	< 0.001

### Associated factors of dietary diversity practice among adolescent

In this study, we assessed factors associated with adequate dietary diversity practice. A separate analysis was conducted for each factor, which was considered in this meta-analysis. Variables assessed with the dietary diversity practice were: maternal education, father education, residence, and income.

**Fig 4:**
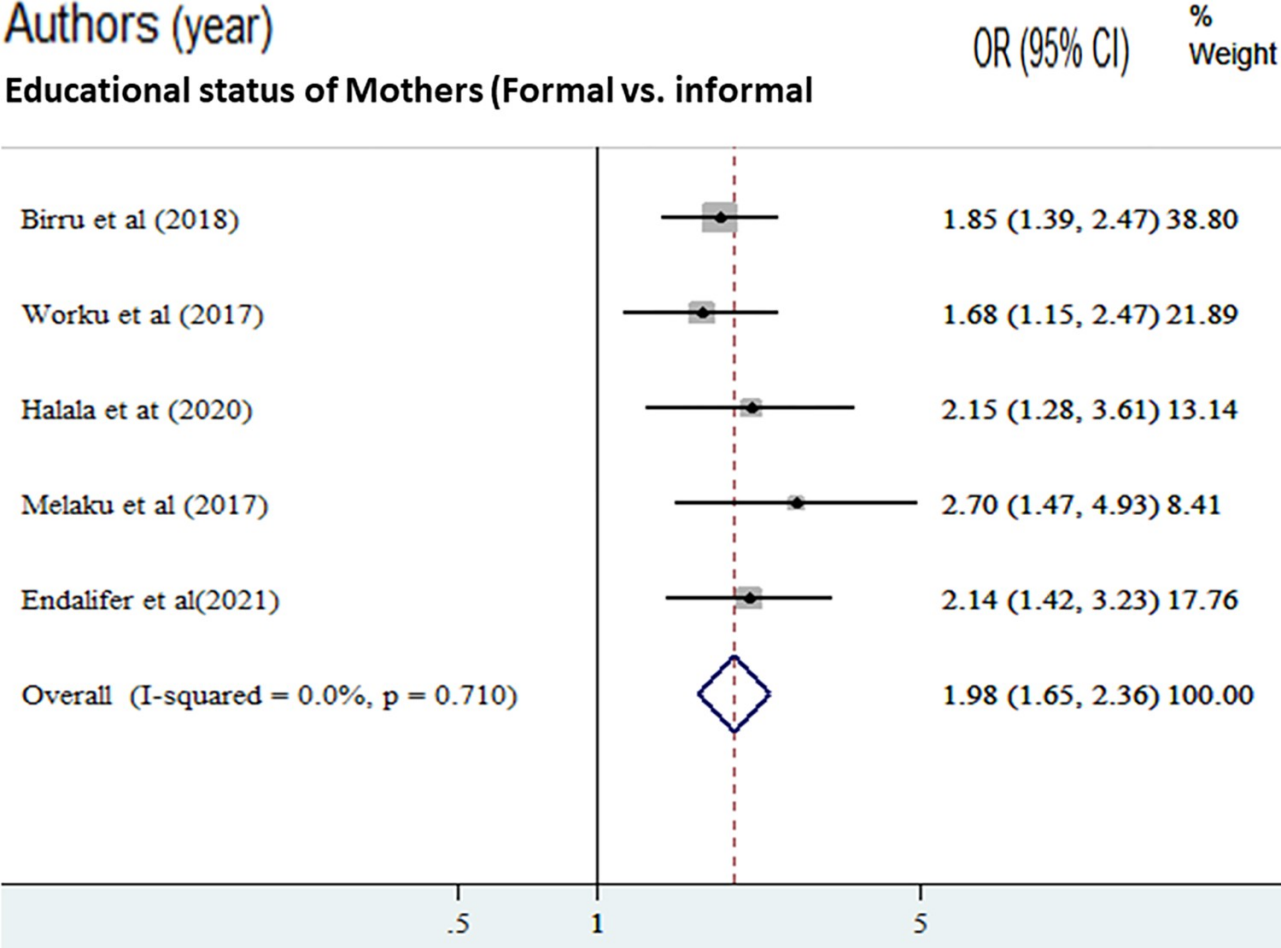
The pooled odds ratio of the association between mothers’ formal education and good dietary diversity practice among adolescents in Ethiopia.

**Fig 5:**
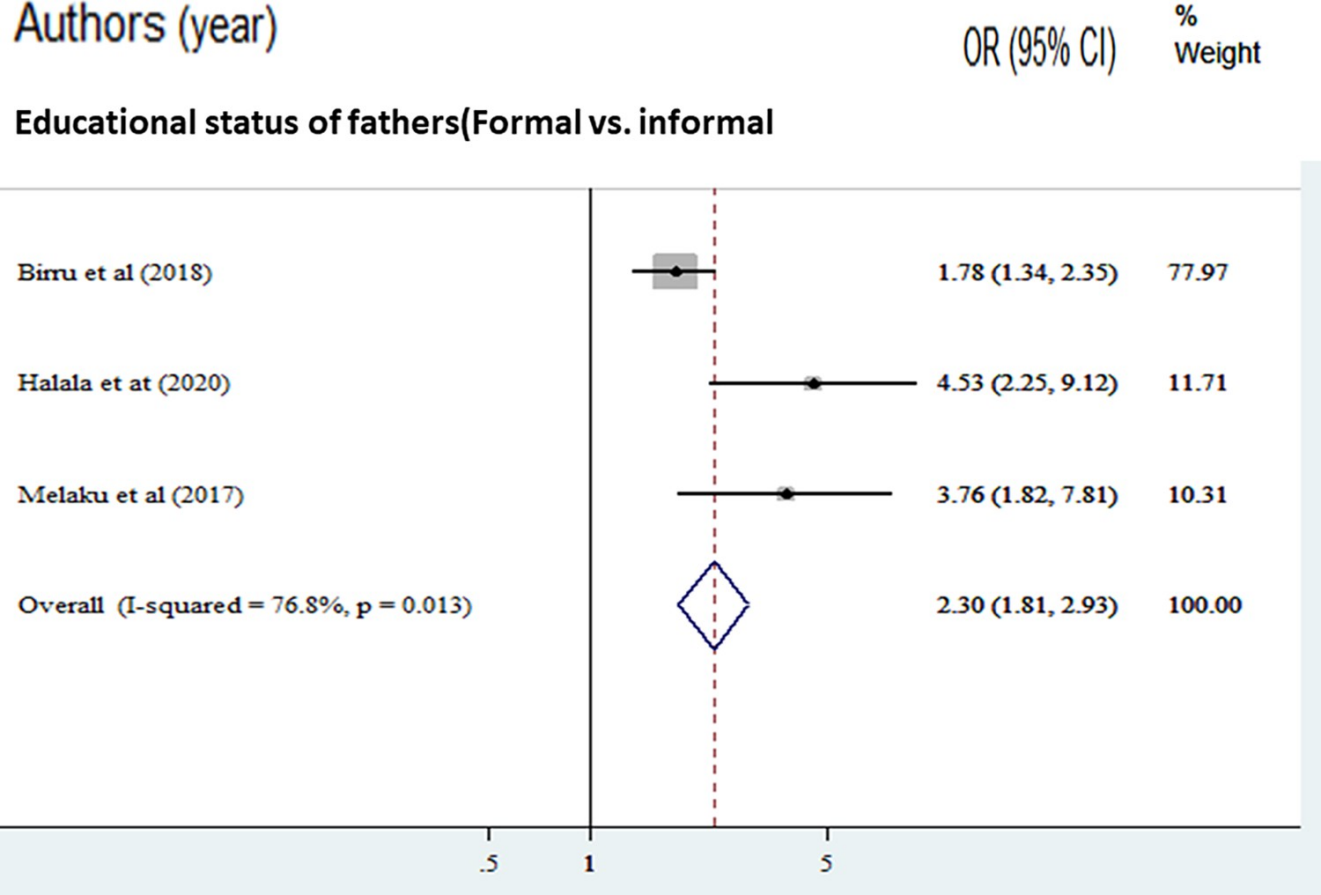
The pooled odds ratio of the association between fathers’ formal education and good dietary diversity practice among adolescents in Ethiopia.

**Fig 6:**
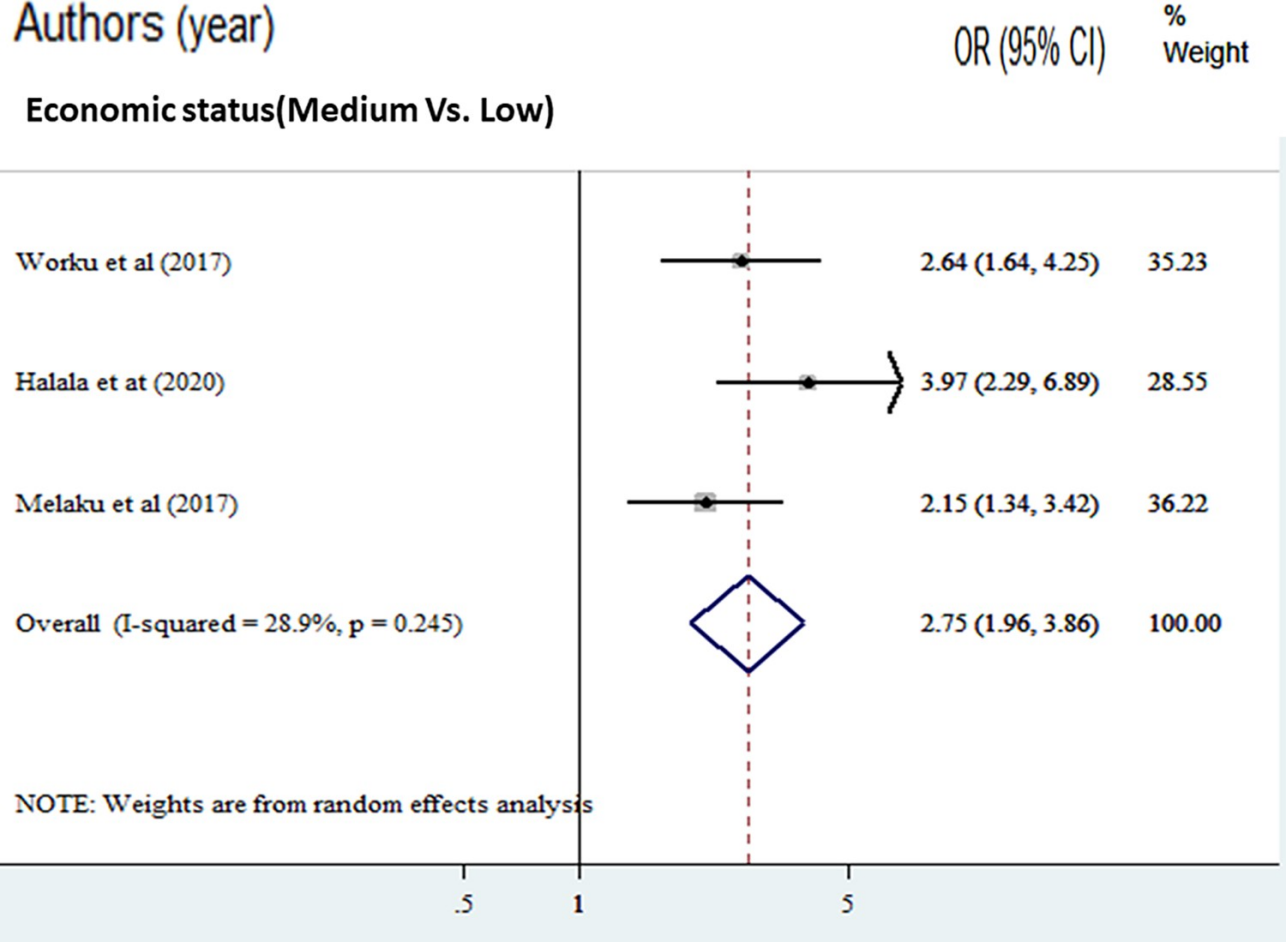
The pooled odds ratio of the association between the medium wealth quintile and good dietary diversity practice among adolescents in Ethiopia.

The odds of adequate dietary diversity practice were 1.98 times higher among mothers’ who had formal education compared with mothers who had no formal education [AOR = 1.98, (95% CI: 1.65, 2.36)]. Overall and within-study design, the value of I ^2^ was low (Fig 4). The odds of adequate dietary diversity practice were 2.30 times higher among fathers with formal education compared with fathers with no formal education [AOR = 2.30, (95% CI: 1.81, 2.93)]. Overall and within the study design, the value of I ^2^ was low (Fig 5). The odds of adequate dietary diversity practice were 2.75 times higher among medium wealth index compared to those adolescents with low wealth index [AOR = 2.75, (95% CI: 1.96, 3.86)]. But, we found that a woman from a household with a high wealth quintile was not associated with dietary diversity practice compared to a poor household (Fig 6).

**Fig 7:**
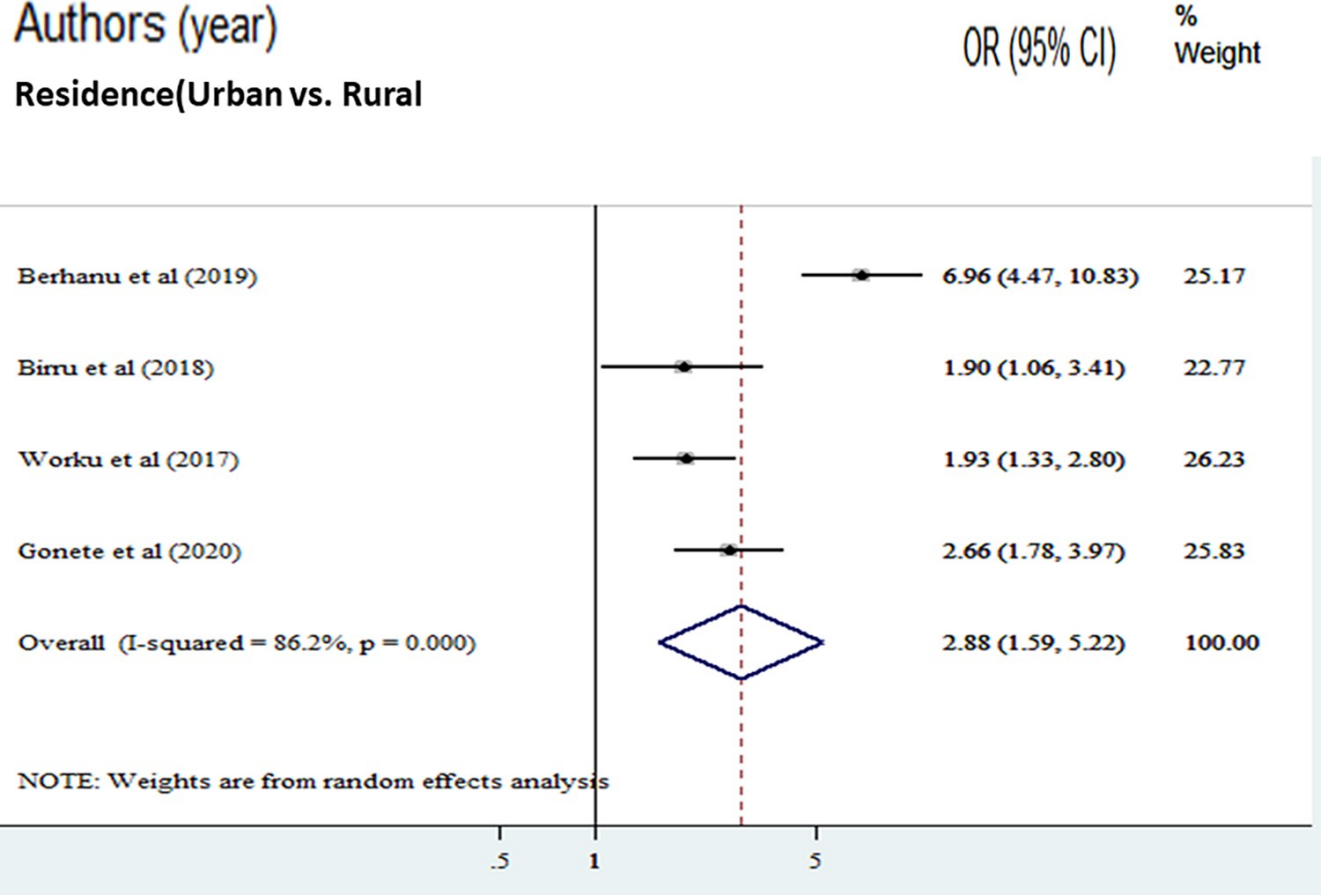
The pooled odds ratio of the association between urban residence and good dietary diversity practice among adolescents in Ethiopia.

The odds of good dietary diversity were 2.88 times higher among rural residence adolescents compared with adolescents who reside in urban [AOR = 2.88, (95% CI: 1.59, 5.22)] (Fig 7).

## Discussion

Good Dietary diversity practice has a significant role for adolescents because the adolescent period is the second window of opportunity. Therefore, the objective of this review was to estimate the pooled prevalence of dietary diversity practice and its associated factors among adolescents in Ethiopia.

According to this meta-analysis, the overall pooled prevalence of dietary diversity practices among adolescents in Ethiopia was 39.24% (95% CI: 30.82, 47.66). The finding of this study reveals that more than one-third of the adolescents consumed adequately diversified food in Ethiopia. This result was consistence with the study conducted in rural Bangladesh (31.5%), Ghana (44%), and Zimbabwe 34% [28–30]. But the pooled prevalence of adequate dietary diversity practice in this meta-analysis was higher than in the study conducted in Dhaka City in Bangladesh (8.7%), Pakistan (13.5%) and Burkina Faso (24.9%) [31, 32]. The possible explanation might be due to difference in the study design, cultural and dietary habit. In addition it might be due to the Pakistan and Burkina Faso studies are not affected by seasonality and include the largest population‐based cohort of adolescent to date.

In addition, this pooled prevalence was lower than the study conducted in Matlab Bangladesh 57%, and eastern Uganda (54.7%) [14, 33]. These discrepancies might be due to differences in sample size, measurement items of adequate dietary diversity and the cut point from one country to another country, seasonal variation of the study period, geographical location variation, and cultural differences. The pooled prevalence of diet diversity practice suggests the nutritional status and reduced quality of diets among adolescents. The finding of this study also indicates poor farming methods in the community.

According to this study finding, adolescents from mothers who had formal education were 1.98 times more likely to have adequate dietary diversity as compared with mothers who had no formal education. This finding was consistent with a study conducted in Bangladesh [14]. This might be due to, education being the main component that affects development and economic growth. And also, educated mothers can easily understand and change into practice nutrition knowledge because home activities and food preparation were covered by females most of the time in the Ethiopia context. And also the mother’s educational status affects the dietary pattern of the adolescents [34].

The pooled odds of adequate dietary diversity among adolescents with fathers who had formal education were 2.3 times higher than adolescents with fathers not had formal education. This finding was in line with the study conducted in Bangladesh [14]. This might be due to when the adolescent had educated parents are more likely to have good nutritional knowledge on the benefit of eating diversified food. Moreover, as a mother’s educational status increases, they will be increased in economic status to fulfil the basic needs of their child. Therefore they are critically seeing their child’s eating practices which might be reflected in their children’s diet quality. The educational status of parents influences the food choice, dietary habits, and dietary patterns of adolescents.

The pooled odds of good dietary diversity practice among adolescents who had a Medium wealth quintile were increased by 2.75 as compared to the Low wealth quintile. This finding was congruent with a study conducted in Bangladesh and eastern Uganda [14, 33]. This may be because when the household asset plays a great role and it is a prerequisite for the practice of dietary diversity. Moreover, the household asset increase the family can buy different types of food. The feeding of fruits, vegetables and animal-source foods increases with household income in many other developing countries [35, 36].

The pooled odds of good dietary diversity practice among adolescents who reside in urban areas were 2.9 times higher than those who live in urban areas. The possible explanation might be when they live in urban can easily access the market to buy food groups.

Even though the analysis had strengths, it has certain limitations: All included articles were institutional-based cross-sectional which may affect the overall point estimate. And also, heterogeneity was not completely fixed in the final random effect model. These findings will have vital implications for program planners, policymakers, and healthcare providers to design nutrition intervention programs for adolescents accordingly.

## Conclusion

The pooled prevalence of good dietary diversity practices among adolescents is low. Being urban residents, medium wealth quintile, mothers’ educational status and fathers’ educational status were found to be associated with good dietary diversity practices among adolescents. The governments and relevant stakeholders should develop and implement effective interventions to increase the prevalence of good dietary diversity practices. More focused interventions should be given to rural residents and adolescents from low economic status. Nutrition education should be given to adolescents and their families to improve the dietary diversity of adolescents. In addition to this, income-generating activities should be implemented to improve the economic status of the household as it affects the dietary diversity score of adolescents.

## Supporting information

S1 File PRISMA check list. (DOCX)

S2 File The datasets used/analysed in this systematic review and meta-analysis. (XLSX)
